# Evaluation of Thio- and Seleno-Acetamides Bearing Benzenesulfonamide as Inhibitor of Carbonic Anhydrases from Different Pathogenic Bacteria

**DOI:** 10.3390/ijms21020598

**Published:** 2020-01-17

**Authors:** Andrea Angeli, Mariana Pinteala, Stelian S. Maier, Bogdan C. Simionescu, Andrea Milaneschi, Ghulam Abbas, Sonia del Prete, Clemente Capasso, Antonella Capperucci, Damiano Tanini, Fabrizio Carta, Claudiu T. Supuran

**Affiliations:** 1 Sezione di Scienze Farmaceutiche, NEUROFARBA Dept., Università degli Studi di Firenze, Via Ugo Schiff 6, 50019 Sesto Fiorentino (Florence), Italy; andrea.angeli@unifi.it (A.A.); andrea.milaneschi@stud.unifi.it (A.M.); fabrizio.carta@unifi.it (F.C.); 2 Centre of Advanced Research in Bionanoconjugates and Biopolymers Department, “Petru Poni” Institute of Macromolecular Chemistry, 700487 Iasi, Romania; pinteala@icmpp.ro (M.P.); smaier@ch.tuiasi.ro (S.S.M.); bcsimion@icmpp.ro (B.C.S.); 3 Polymeric Release Systems Research Group, Polymers Research Center, “Gheorghe Asachi” Technical University of Iasi, 700050 Iasi, Romania; 4 Department of Biological Sciences and Chemistry, University of Nizwa, Birkat Al-Mauz, P.O.Box 33, Nizwa-616, Sultanate of Oman; abbashej@unizwa.edu.om; 5 Istituto di Bioscienze e Biorisorse, CNR, Via Pietro Castellino 111, 80131 Napoli, Italy; sonia.delprete@unifi.it (S.d.P.); clemente.capasso@ibbr.cnr.it (C.C.); 6 Department of Chemistry “Ugo Schiff”, Università degli Studi di Firenze, Via della Lastruccia 13, I-50019 Sesto Fiorentino (Florence), Italy; antonella.capperucci@unifi.it (A.C.); damiano.tanini@unifi.it (D.T.)

**Keywords:** carbonic anhydrase, inhibitors, metalloenzymes, selenium, *Vibrio cholera*, *Burkholderia pseudomallei*, *Mycobacterium tuberculosis*, salmonella

## Abstract

A series of 2-thio- and 2-seleno-acetamides bearing the benzenesulfonamide moiety were evaluated as Carbonic Anhydrase (CA, EC 4.2.1.1) inhibitors against different pathogenic bacteria such as the *Vibrio cholerae* (VchCA-α and VchCA-β), *Burkholderia pseudomallei* (BpsCA-β and BpsCA-γ), *Mycobacterium tuberculosis* (Rv3723-β) and the *Salmonella enterica serovar Typhimurium* (StCA2-β). The molecules represent interesting leads worth developing as innovative antibacterial agents since they possess new mechanism of action and isoform selectivity preferentially against the bacterial expressed CAs. The identification of potent and selective inhibitors of bacterial CAs may lead to tools also useful for deciphering the physiological role(s) of such proteins.

## 1. Introduction

Infectious diseases caused by human pathogens namely bacteria, fungi and virus are among the major challenges for humankind. A large number of bacteria, in recent years, showed increasing resistance to the more common antibiotics clinically used [1,2,3]. Despite the progresses in fundamental knowledge on various pathogens, the current chemotherapy still is unsatisfactory due to limited efficacy, long-term treatment, drug resistance and undesired side effects. Thus, there is an urgent need for the development of new and efficient drugs against human affecting pathogens. Recently, the inhibition of bacterial Carbonic Anhydrases (CAs EC 4.2.1.1) was demonstrated to influence both growth and pathogenicity of microorganisms [4,5,6], and this allows to properly define a new approach for obtaining anti-infective agents with a new mechanism of action when compared to classical antibiotics [7]. These essential enzymes are a superfamily of metalloenzymes which catalyze the reversible hydration of CO_2_ in H_2_CO_3_ [8]. To date, seven genetically distinct families were reported and were named as α- (present in vertebrates, protozoa, algae, cytoplasm of green plants, and in many gram-negative Bacteria), β- (in both gram-negative and -positive Bacteria, mono and dicotyledons plants, fungi and Archaea), γ- (Bacteria, cyanobacteria and Archaea), δ-, ζ-, θ- (in marine diatoms) and ɳ- CAs (protozoa belonging to the *Plasmodium spp*) [9,10,11,12].

Since our proof-of-concept on organochalcogen derivatives having interesting inhibition activity against some bacterial expressed CAs [13] further successful investigations were carried-out [10,14]. The study herein proposed is placed on this spot with the intent to further investigate such a class of compounds for their inhibition properties against selected bacterial expressed CAs.

## 2. Results and Discussion

### 2.1. Chemistry

**Figure 1:**
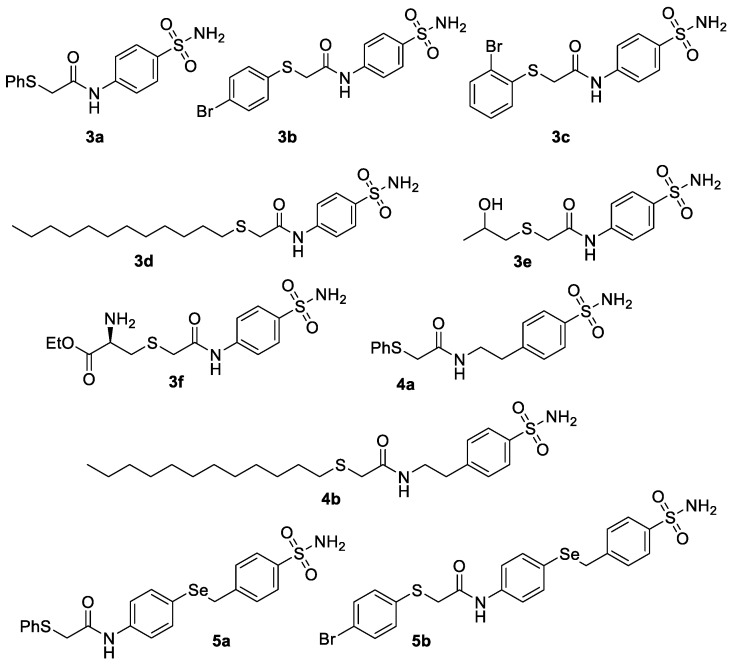
Sulfur-containing acetamides bearing the benzenesulfonamide moiety.

We focused our attention onto the study of substituted 2-thio- and 2-seleno-acetamides bearing the benzenesulfonamide moiety. We sought to employ 2-chloroacetamides **2a**–**c**, obtained according to previously reported procedures [15], as precursors of the synthesis of the polyfunctionalised target compounds through the reaction with suitable sulfur- and selenium-containing nucleophiles. As reported previously by our group [16], this methodology enables the synthesis of differently substituted sulfur-containing *N*-(4-sulfamoylphenyl)acetamides as outlined in Figure 1.

**Figure 2:**
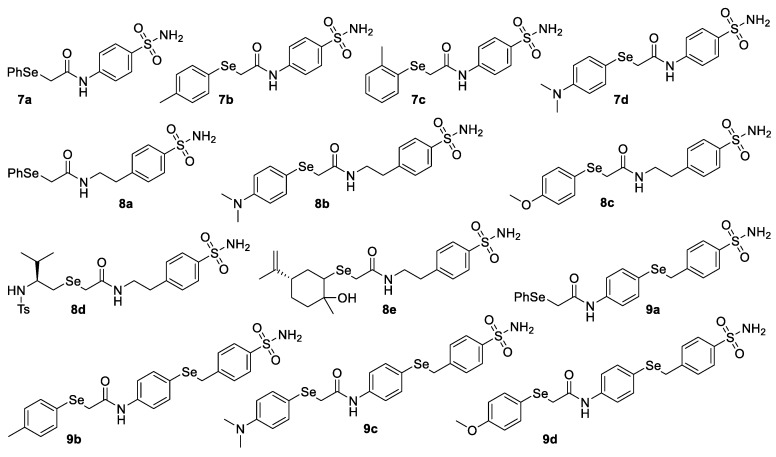
Selenium-containing acetamides bearing the benzenesulfonamide moiety.

In order to enlarge the library of these novel chalcogen-containing molecules as potential CA inhibitors, we also synthetized the selenium-containing analogues of compounds **3**–**5**. Diselenides were employed as precursors of selenolate anions and in situ treated with 2-chloroacetamides **2a**–**c**. Diselenides **6b**–**d**, bearing p-methyl-, o-methyl-, and p-(*N,N*-dimethylamino)-substituted aromatic rings, were efficiently converted into the corresponding 2-(arylseleno)acetamides **7b**–**d** in good yields under the same reaction conditions (Figure 2).

**Scheme 1:**
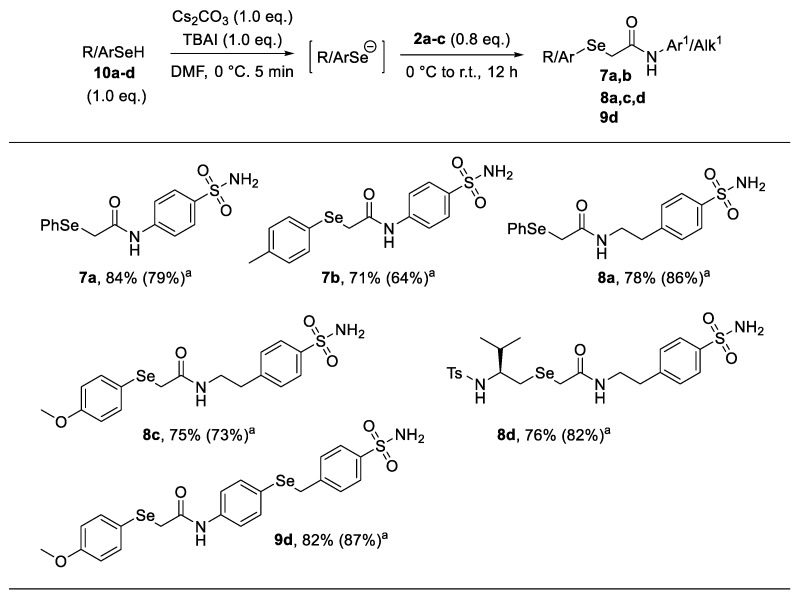
Alternative route for the synthesis of selenium-containing acetamides bearing the benzenesulfonamide moiety exploiting the reactivity of selenols **10a**–**d**. Yields refer to isolated products. ^a^ Yields refer to ref. [16].

Finally, we further enlarge the scope of this procedure with variously substituted diselenides allowing the synthesis of organoselenides **8a**–**c** from diselenides **6a**–**c** and diselenides **6a,b,d,e** gave *N*-(4-(benzylselanyl)phenyl)acetamides **9a**–**d**, bearing two selenated moieties onto the same molecular skeleton (Figure 2). Additionally, on the basis of our recent findings on the reactivity of selenols with elecrophiles [17,18,19,20], we also evaluated an alternative and milder approach to 2-seleno-acetamides **7**–**9**. Thus, aryl- and alkyl-selenols **10a**–**d** were efficiently deprotonated by using a Cs_2_CO_3_/TBAI system [21,22] and then treated with 2-chloroacetamides **2a**–**c** to afford the corresponding selenium-containing acetamides **7**–**9** in good yield (Scheme 1). Notably, owing to the mildness of the reaction conditions, this methodology could represent a valuable alternative route for the synthesis of 2-seleno-acetamides bearing labile moieties, poorly stable under strong reducing conditions. All obtained compounds were characterized by means of ^1^H-NMR, ^13^C-NMR, ^77^Se-NMR and mass spectra analysis and were in agreement with the previously reported data [16].

### 2.2. Carbonic Anhydrase Inhibition

Our main interest was to investigate structure-activity relationship (SAR) features of our **3a-f**, **4a-b**, **5a-b**, **7a-c**, **8a-c**, **9a-d** compounds underpinning the inhibition against different bacterial CA isoforms such as *Vibrio cholerae* (VchCA-α and VchCA-β), *Burkholderia pseudomallei* (BpsCA-β and BpsCA-γ), *Mycobacterium tuberculosis* (Rv3723-β) and *Salmonella enterica serovar Typhimurium* (StCA2-β). The inhibition studies were performed by means of the stopped-flow carbon dioxide hydration assay [23], compared to the standard and clinically used CAI acetazolamide (**AAZ**) (Table 1).

The following SARs can be drawn out of the data reported in Table 1:

(i) The inhibition profile of the only α-CA isoform reported in this study (i.e., the VchCA-α) was deeply influenced by compounds herein tested. The range of K_I_ values spanned from low to high nanomolar range (K_I_ 2.2–902.2 nM) and the compounds **3d** and **4b** bearing the nonanethiol tail were devoid of any activity (Table 1). The insertion of the bromine atom within the thiophenol moiety as in compounds **5b** increased up to 18 folds the inhibition activity when compared to **5a** which does not have any halogen (K_I_ 829.1 to 45.8 nM). An interesting SAR case was the replacement of the sulfur atom in **3a-b** with selenium one to afford compounds **7a-b**. Such an isosteric substitution increased the inhibition potency up to low nanomolar values (K_I_s 49.9, 46.7 and 2.2, 7.7 nM respectively).

(ii) The second isoform here tested from the Gram-negative bacterium *Vibrio Cholerae*, VchCA-β, was less inhibited when compared to the α- as the associated K_I_ values were comprised between high nanomolar to micromolar range (K_I_ 82.9–7985 nM). On the other hand, compound **3f** did not show any inhibitory activity against this isoform. The replacement of sulfur with selenium atom by compounds **3a** and **7a**, also for this isoform, lead to increase the activity over 48 times (K_I_ 4648 nM for **3a** and 96.3 nM for **7a**). Conversely, such a replacement was not effective for compounds **5a** and **9a** as the inhibition constants were perfectly superimposable (K_I_s 829.1 and 902.2 nM, respectively) or were decreased as for compounds **4a** and **8a** (700 and 6590 nM respectively).

(iii) All compounds herein considered had not effects on the BpsCA-β except for derivative **7a**, which was a weak inhibitor (K_I_ 4094 nM).

(iv) As for the BpsCA-γ the replacement of sulfur atom with selenium one (compounds **3a** and **7a**) lead to increase activity over 100 folds (K_I_ 4168 and 303.8 nM, respectively). Moreover, for the series **9a-d**, substituents on the aromatic ring proved to be detrimental for the inhibition potency (K_I_ 449.7–10000 nM). Substitutions on the aromatic rings within the series **7a-d** proved to be crucial for the activity as reported for the ortho CH_3_ in **7c** which resulted in loss of the inhibition potency. The BpsCA-β was inhibited only from compound **7a** (K_I_ 4094 nM).

(v) The *Mycobacterium tuberculosis* β-CA Rv3273 was weakly or not inhibited at all by compounds here reported. In general, the series **9a-d** and **7a-d** showed the same inhibition features of BpsCA-γ.

(vi) Finally, the StCA2-β from the bacterial pathogen *Salmonella enterica serovar Typhimurium* was deeply influenced by these compounds. As VchCA-α the range of inhibition spanned from low to high nanomolar range (K_I_ 7.5–88.1 nM) and for compounds **7b**, **9a** no activity was recorded. Within the **3a-f** series the bromine atom proved to be essential for the potency (**3b-c**), indeed, the activity increased 9 times than the analog without substituent (**3a**) or different derivatives as in **3d-f**. In analogy, as mentioned above, compound **5b** with bromine atom showed an inhibition constant 100 folds better than **5a** (K_I_ 8.2–865 nM, respectively). StCA2-β was the only isoform here reported where the replacement of sulfur atom of compound **3a** with selenium one (**7a**) did not show improvement in the inhibition activity. An interesting case was for the series **9a-d** where the substituents on aromatic ring were fundamental for the inhibition of this isoform showing no activity for compound **9a** without them.

Overall, the substituted 2-thio- and 2-seleno-acetamides bearing the benzenesulfonamide revealed interesting inhibition features and selectivity profiles. Compounds **3f** did not show inhibition against the β CA isoforms except for the StCA2-β, which was weakly inhibited and thus proving this molecule to possess a good selectivity for the α isoforms such as the VchCA-α. Derivatives **4b** and **5b** did not show any activity against the bacterial isoforms of *Burkholderia pseudomallei* and *Mycobacterium tuberculosis* proving good inhibitors of the β isoform of Salmonella. In general, series **7a-c** had selectivity for α isoform of Vibrio cholera. Finally, series **8a-e** and **9a-d** did not show particular potency of inhibition for BpsCA-β and γ but activity against the VchCA isoforms.

## 3. Materials and Methods

### 3.1. General

Anhydrous solvents and all reagents were purchased from Sigma-Aldrich, Alfa Aesar and TCI. The solvents used in MS measures were acetone, DMSO, acetonitrile (Chromasolv grade), purchased from Sigma-Aldrich (Milan, Italy), and mQ water 18 MΩ, obtained from Millipore’s Simplicity system (Milan, Italy).

### 3.2. Chemistry

Characterization of compounds **3a-f**, **4a-b**, **5a-b**, **7a-c**, **8-e** and **9-a-d** was reported earlier by our group [16]. β-mercapto alcohol 1e [24], alkyl selenols [22] and aryl selenols [25] were prepared according previously reported procedures.

Typical procedure for the synthesis of 2-seleno-acetamides through alkylation of selenols:

A solution of selenol (1.0 mmol) in dry DMF (5 mL) was cooled under inert atmosphere at 0 °C and treated with Cs_2_CO_3_ (326 mg, 1.0 mmol) and TBAI (369 mg, 1.0 mmol). Then, a DMF solution (1 mL) of the suitable 2-chloroacetamide 2a-c (0.8 mmol) was added and the mixture was allowed to warm to r.t. and stirred for 12 h. Afterwards, the reaction was treated with saturated NH_4_Cl solution (2 mL), extracted with EtOAc (10 mL) and washed with water (2 × 10 mL) and brine (2 × 10 mL). The organic phase was dried over Na_2_SO_4_, the solvent was evaporated under vacuum and the crude product was purified by flash column chromatography or precipitated from EtOAc/petroleum ether to yield substituted 2-selenoacetamides 7,8,9.

### 3.3. Carbonic Anhydrase Inhibition

An Applied Photophysics stopped-flow instrument has been used for assaying the CA catalyzed CO_2_ hydration activity [23]. Phenol red (at a concentration of 0.2 mM) has been used as indicator, working at the absorbance maximum of 557 nm, with 20 mM Hepes (pH 7.5 for the α-CAs) or TRIS (pH 8.3 for the β-CAs) as buffers, and 20 mM Na_2_SO_4_ (for maintaining constant the ionic strength), following the initial rates of the CA-catalyzed CO_2_ hydration reaction for a period of 10–100 s. The CO_2_ concentrations ranged from 1.7 to 17 mM for the determination of the kinetic parameters and inhibition constants. For each inhibitor, at least six traces of the initial 5% to 10% of the reaction have been used for determining the initial velocity. The uncatalyzed rates were determined in the same manner and subtracted from the total observed rates. Stock solutions of inhibitor (0.1 mM) were prepared in distilled-deionized water and dilutions up to 0.01 nM were done thereafter with the assay buffer. Inhibitor and enzyme solutions were preincubated together for 15 min at room temperature prior to assay, in order to allow for the formation of the E-I complex. The inhibition constants were obtained by non-linear least-squares methods using PRISM 3 and the Cheng–Prusoff equation, as reported earlier [26,27,28,29,30,31], and represent the mean from at least three different determinations. All CA isoforms were recombinant ones obtained in-house as reported earlier [26,27,28,29,30,31].

## 4. Conclusions

In conclusion, we investigated a series of 2-thio- and 2-seleno-acetamides bearing the benzenesulfonamide for their inhibition against different carbonic anhydrase isoforms from several pathogenic bacteria such as *Vibrio cholera*, *Burkholderia pseudomallei*, *Mycobacterium tuberculosis* and *Salmonella enterica serovar Typhimurium*. Regarding their well-known zinc binding group, the primary sulfonamide, these compounds showed an interesting inhibition activity with different selectivity profile. Among them, compounds **3f** and **7a-c** had a good selectivity for VchCAα. Derivatives **4b** and **5b** proved good inhibitors of the β isoform of Salmonella and, finally, series **8a-e** and **9a-d** showed activity against both VchCA isoforms. Thus, this series of compounds might be considered promising lead compounds for obtaining more effective and selective CAIs targeting different bacterium isoforms and as useful pharmacological tools for understanding the physiological role(s) of these under-investigated enzymes.

## Figures and Tables

**Table 1:** Inhibition data against bacterial enzymes VchCAα-β, BpsCAβ-γ, StCA2-β and Rv3273-β of compounds **3a-f**, **4a-b**, **5a-b**, **7a-c**, **8a-c**, **9a-d** and acetazolamide (**AAZ**) by a stopped flow CO_2_ hydrase assay [23].

K_I_ (nM) *						
Cmp	VchCA-α	VchCA-β	StCA2-β	BpsCA-β	BpsCA-γ	Rv3273-β
**3a**	49.9	4648	797.9	>10,000	4168	2779
**3b**	46.7	700.0	85.6	>10,000	2750	1833
**3c**	33.5	865.0	84.1	>10,000	404.7	269.8
**3d**	>10,000	581.4	752.7	>10,000	3819	2546
**3e**	44.7	6651	77.7	>10,000	321.5	214.3
**3f**	84.9	>10,000	482.8	>10,000	>10,000	>10,000
**4a**	51.1	700.0	86.7	>10,000	3515	2343
**4b**	>10,000	7187	881.1	>10,000	>10,000	>10,000
**5a**	829.1	6225	865.0	>10,000	1517	1011
**5b**	45.8	580.7	8.2	>10,000	>10,000	>10,000
**7a**	2.2	96.3	814.7	4094	303.8	202.5
**7b**	7.7	762.2	>10,000	>10,000	424.2	282.8
**7c**	41.7	6210	451.3	>10,000	>10,000	>10,000
**8a**	44.4	6590	86.9	>10,000	2250	1500
**8b**	85.6	607.8	550.0	>10,000	3634	2422
**8c**	3.2	85.0	9.3	>10,000	>10,000	>10,000
**8d**	62.3	828.1	9.1	>10,000	>10,000	>10,000
**8e**	6.1	308.9	8.9	>10,000	>10,000	>10,000
**9a**	902.2	7468	>10,000	>10,000	449.7	299.8
**9b**	88.6	729.5	27.3	>10,000	4172	2781
**9c**	730.3	7985	820.0	>10,000	3656	2437
**9d**	8.9	82.9	7.5	>10,000	>10,000	>10,000
**AAZ**	6.8	451	89.0	745	149	104

* Mean from 3 different assays, by a stopped flow technique (errors were in the range of 5–10% of the reported values).

## Abbreviations

CA	Carbonic Anhydrase
CAI	Carbonic Anhydrase Inhibitors
